# Chemotherapeutic Drugs Interfere with Gene Delivery Mediated by Chitosan-Graft-Poly(ethylenimine)

**DOI:** 10.1371/journal.pone.0126367

**Published:** 2015-05-11

**Authors:** Wing-Fu Lai, Marie C. Lin

**Affiliations:** 1 School of Medicine, Shenzhen University, Shenzhen, China; 2 Department of Mechanical Engineering, The University of Hong Kong, Hong Kong Special Administrative Region, China; Universidad de Castilla-La Mancha, SPAIN

## Abstract

Combined chemo-gene therapy is one of the treatment modalities that have attracted extensive research interests; however, there is little information regarding the influence of drug application on gene transfer. This study bridges this gap by examining how chemotherapeutic drugs (teniposide, *cis*-diamminedichloroplatinum(II) and temozolomide) interfere with polyplex formation and transfection of chitosan-graft-poly(ethylenimine). Our results indicate that the degree of drug interference varies with the mechanism of drug action, with the transgene expression being severely suppressed when the plasmid is co-delivered with *cis*-diamminedichloroplatinum(II) or teniposide but not temozolomide. In addition, the interference with transfection by drugs varies with different gene/drug co-formulations. This is the first study to evidence that, though combined chemo-gene therapy has therapeutic potential, some chemotherapeutic drugs may reduce the treatment efficiency of gene therapy.

## Introduction

Chemotherapy is one of the most widely adopted regimes in cancer treatment, yet its efficiency has been impeded by drug resistance developed in cancer cells [1–6]. One of the emerging approaches to coping with drug resistance is combined chemo-gene therapy [7,8], which aims at enhancing the action of chemotherapeutic drugs and re-sensitizing drug-resistant cells to chemotherapy. The promising potential of combined chemo-gene therapy was evidenced *in vitro* by an earlier study in human hepatocellular carcinoma HepG2 cells [9]. In the study, the cell treatment was mediated by double-walled microspheres, which comprised poly(D,L-lactic-co-glycolic acid) (PLGA) cores and poly(L-lactic acid) (PLLA) shell layers. The microspheres delivered doxorubicin concomitantly with chitosan/DNA nanoparticles containing the p53-encoding plasmid. The combined treatment was shown to enhance cancer cell death more efficiently than treatment with either the polyplexes or the drug [9]. In addition, p53 over-expression could activate caspase-3, thereby enhancing the anti-proliferative effect of doxorubicin in HepG2 cells [9]. More recently, transfection of *cis*-diamminedichloroplatinum(II) (CDDP)-resistant SKOV3/DDP cells with a *p53* gene/MDM2-siRNA plasmid was found to reduce the protein expression of MDR1/P-gp while increasing p53, PUMA and NOXA expression [10]. Such a change in protein expression increased the sensitivity of the cells to CDDP by suppressing apoptotic resistance and enhancing intracellular platinum accumulation. This reduced the protein expression of HIF-1, VEGF, MMP-9 and MMP-2, and finally inhibited cell invasion and migration [10]. Apart from *in vitro* studies, the potential of chemo-gene therapy was supported by an *in vivo* study, which used a human adenovirus type 5 (dE1/E3) containing the human *ABCA10* transgene under the control of the CMV promoter to enhance the effect of CDDP in lung cancer treatment [11]. The increase in the expression of ABCA proteins augmented the therapeutic effect of CDDP. This, along with other evidence in the literature [9–14], has illustrated the prospects of combined chemo-gene therapy in future cancer treatment.

Despite the prospects mentioned above, there is still a limited understanding of the interference with gene transfer by chemotherapeutic drugs. This study has used chitosan-graft-poly(ethylenimine) (CP) to examine the effect of three chemotherapeutic drugs [teniposide (VM-26), CDDP and temozolomide (TMZ)] on polyplex formation and transfection. CP copolymers are one of the chitosan derivatives that have been extensively studied for non-viral gene delivery. An earlier study has reported that the transfection efficiency of the CP copolymers is comparable to that of Fugene HD in B16, U87, HeLa and C666-1 cells [15]. A similar observation of the high transfection efficiency of CP was also reported by Hu and co-workers [16], who found that CP not only has a higher transfection efficiency than PEI 25 kDa *in vitro* but can also effectively deliver the *CCL22* gene to reduce the tumor growth rate *in vivo* [16]. Regarding the potential of CP in cancer treatment, this copolymer has been used as a non-viral vector in this study to evaluate the interference with gene delivery by chemotherapeutic drugs.

## Materials and Methods

### 2.1 Plasmid preparation

The plasmid pEGFP-N1 was purchased from BD Biosciences (San Jose, CA). It was transformed into competent DH5α cells, and plated onto LB plates supplemented with 100 μg/mL ampicillin. Afterwards, it was purified with the Plasmid Giga Kit (Quiagen, Valencia, CA) according to the manufacturer's guidelines. The quality and quantity of the purified plasmid were analyzed by measuring its optical densities at 260 and 280 nm.

### 2.2 Cell culture

Human glioblastoma U87 cells were purchased from ATCC (American Type Culture Collection). The cells were cultured in minimum essential medium (MEM) containing 10% fetal bovine serum, penicillin (100 units/mL) and streptomycin (100 μg/mL).

### 2.3 Depolymerization of chitosan

Chitosan was depolymerized according to a method modified from Mao, *et al*. [17]. Briefly, 1 g of chitosan was suspended in 98 mL of distilled water. 1 mL of 99% acetic acid and 4 mL of 0.15 M sodium nitrite solution were then added. The reaction mixture was allowed to stay at 25°C for 3 hours. Afterwards, the reaction mixture was filtered, and its pH was adjusted to 8.0. The reaction mixture was centrifuged at 3500 × g for 15 minutes, and the pellet was collected. The pellet was washed with distilled water thrice prior to lyophilization.

### 2.4 Polymer synthesis

The depolymerized chitosan (0.4 g) was dissolved in 4 mL of 0.1% acetic acid. 30 mL of degassed dimethyl sulfoxide (DMSO), 60 μL of triethylamine and 0.6 g of 1,1’-carbodiimidazole (CDI) were added. After 4 hours, poly(ethylenimine) (PEI) (Mw = 1.3 kDa) in DMSO (0.14 g/mL) was added drop-wise. The reaction mixture was stirred overnight before dialysis and lyophilization.

### 2.5 Transfection with gene/drug co-formulations

**Fig 1:**
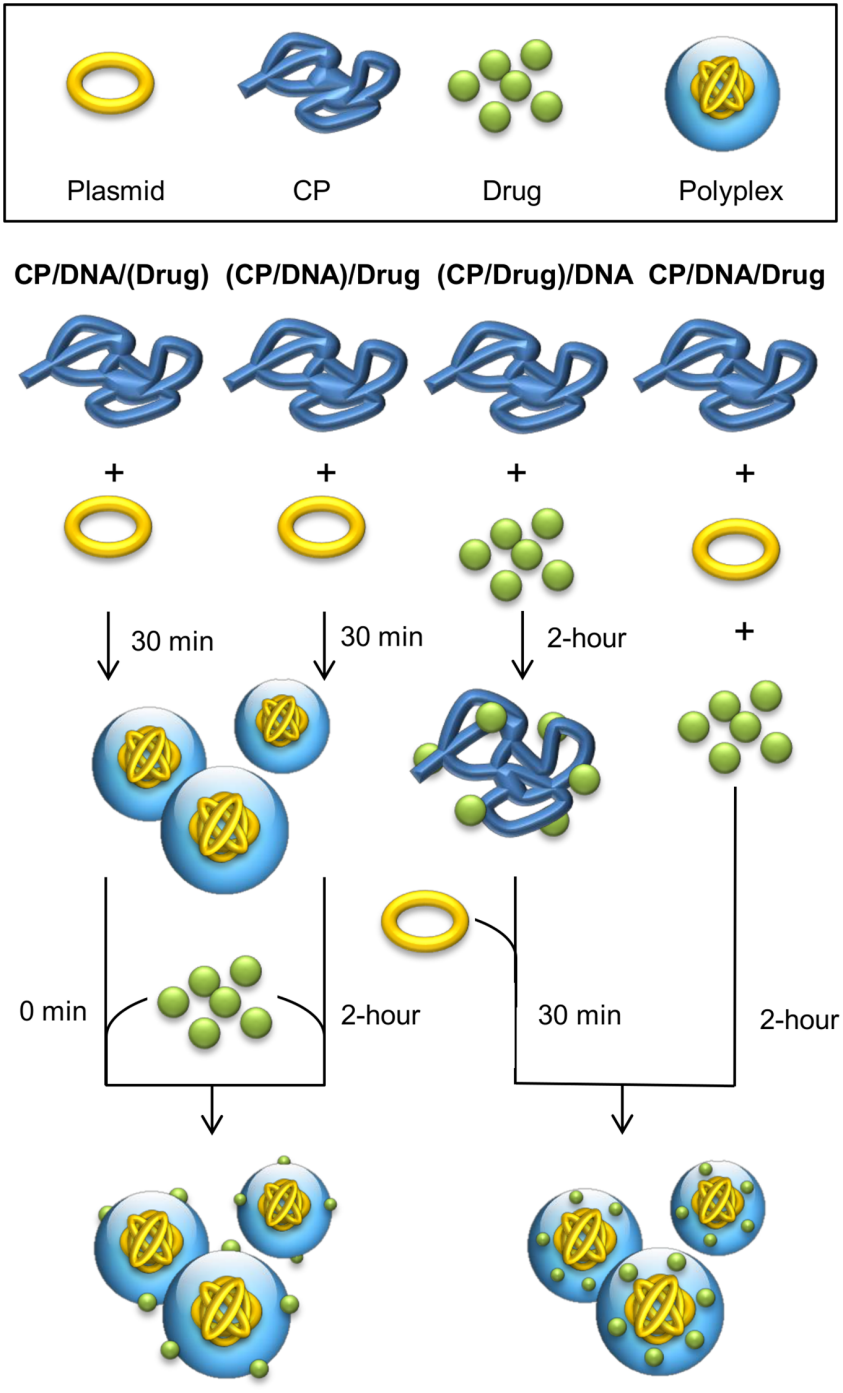
Schematic representations of different approaches of drug/gene co-formulation.

The polymer solution was prepared by solubilizing CP in distilled water at a concentration of 5 μg/μL. VM-26, CDDP and TMZ were dissolved in distilled water at desired concentrations to obtain different drug solutions. The DNA solution was prepared by dissolving pEGFP-N1 in Tris/EDTA buffer at a concentration of 1 μg/μL. Different methods were then adopted for polyplex preparation (Fig 1). In all formulations, the polymer/DNA (m/m) ratio was taken as 20/1.

#### CP/DNA/(Drug)

The polymer and DNA solutions were vortexed for 30 seconds, and then left at 37°C for 30 minutes. Afterwards, the polyplex solution was mixed with an aqueous solution of the chosen drug right before transfection.

#### CP/DNA/Drug

The polymer, drug and DNA solutions were vortexed for 30 seconds, and then left at 37°C for 2 hours

#### (CP/DNA)/Drug

The polymer and DNA solutions were vortexed for 30 seconds, and then left at 37°C for 30 minutes. Afterwards, the polyplex solution was mixed with an aqueous solution of the chosen drug, and left at 37°C for 2 hours.

#### (CP/Drug)/DNA

The polymer and drug solutions were mixed, and left at 37°C for 2 hours. Afterwards, the mixture was mixed with the DNA solution, and left at 37°C for 30 minutes.

24 hours before transfection, U87 cells were seeded in a 24-well plate at a density of 20,000–50,000 cells per wel1. During transfection, the cell culture medium was replaced with 0.5 mL of fresh culture medium. The formulation prepared above was added to each well, and the plate was incubated at 37°C for 5 hours. After that, the transfection medium was removed and replaced with fresh culture medium. The cells were incubated at 37°C for 48 hours before further examination.

### 2.6 Transfection of pre-treated cells

U87 cells were seeded in a 24-well plate at a density of 20,000–50,000 cells per wel1, and incubated at 37°C for 24 hours. After that, an aqueous solution of the chosen drug was added to each well until the desired drug concentration was reached. The cells were incubated at 37°C for 2 hours. Then the medium was replaced with 0.5 mL of fresh culture medium. Polyplexes were prepared by first mixing the polymer and DNA solutions together at a polymer/DNA (m/m) ratio of 20/1, followed by an incubation of the solution mixture at 37°C for 30 minutes. The polyplex solution was added to each well, and the plate was incubated at 37°C for 5 hours. After that, the transfection medium was removed and replaced with fresh culture medium. The cells were incubated at 37°C for 48 hours before further examination.

### 2.7 Quantification of transfected cells

The expression of EGFP in transfected cells was monitored by confocal fluorescence microscopy after 48 hours of post-transfection incubation. The percentage of transfected cells was an average of the number of EGFP-expressing cells in five fields randomly selected at 200 X magnification. The experiment was replicated thrice.

### 2.8 Cytotoxicity tests

The 3-(4,5-dimethylthiazol-2-yl)-2,5-diphenyltetrazolium bromide (MTT) assay was performed as previously described [18]. In brief, after 48 hours of post-transfection incubation, 20 μL of the filtered MTT reagent (0.5 mg/mL) was added to each well. The cells were incubated at 37°C for 5 hours. The unreacted reagent was then aspirated, and the cells were washed with PBS. The violet crystals in each well were dissolved in 100 μL of DMSO. The color intensity was measured by an ELISA reader at a wavelength of 595 nm. Cell viability (%) in each well was determined by dividing the absorbance value (A_595_) of the test well by the A_595_ value of the control well, followed by a multiplication of the quotient by 100.

## Results

### 3.1 Effects of gene/drug co-formulation

**Fig 2:**
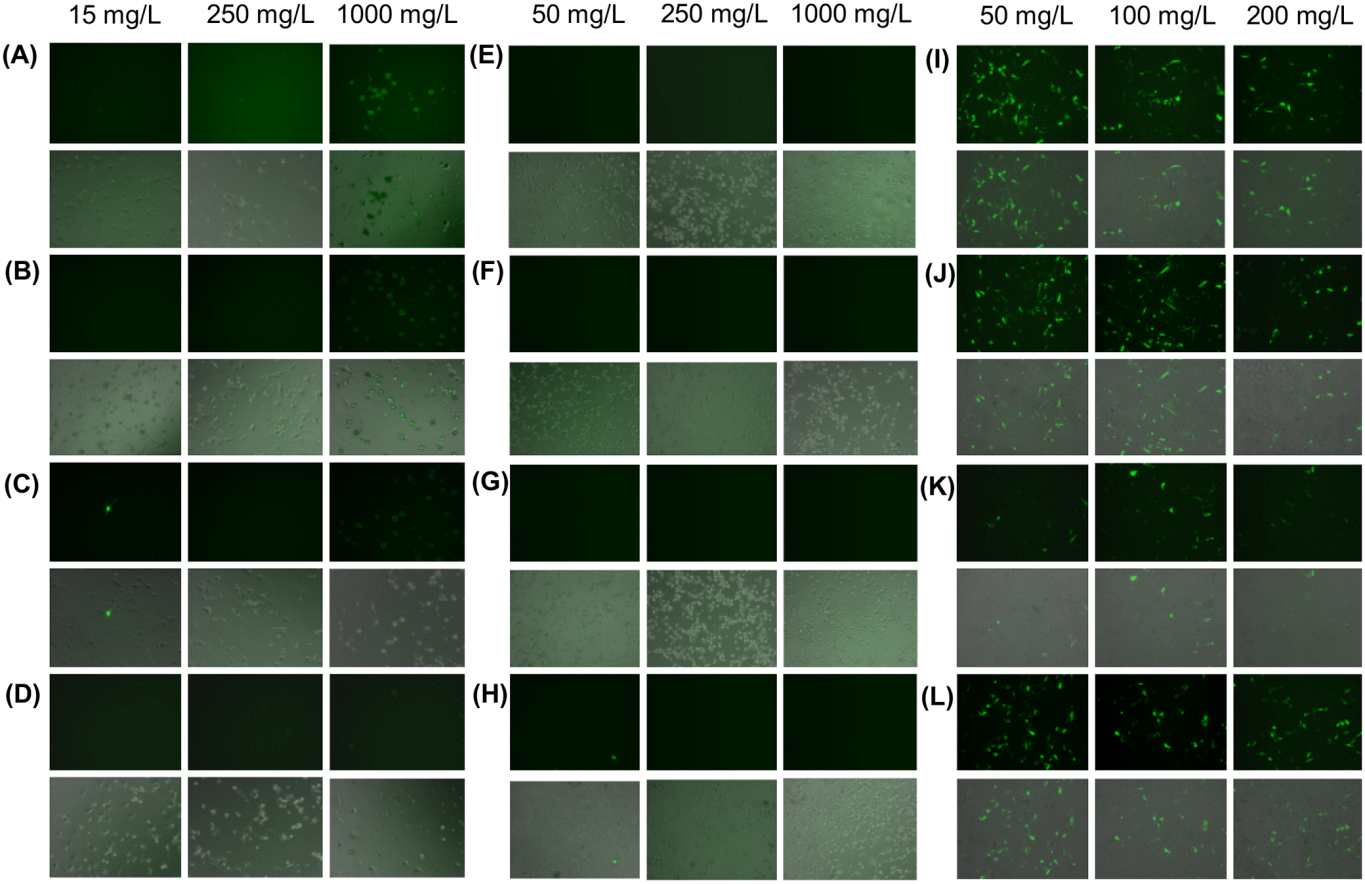
Representative EGFP fluorescence and combined phase-fluorescence micrographs of U87 cells treated with different drug/gene co-formulations. Those co-formulations were (A, E, I) CP/DNA/(Drug), (B, F, J) CP/DNA/Drug, (C, G, K) (CP/DNA)/Drug and (D, H, L) (CP/Drug)/DNA. They were prepared with various concentrations of (A-D) VM-26, (E-H) CDDP and (I-L) TMZ.

**Fig 3:**
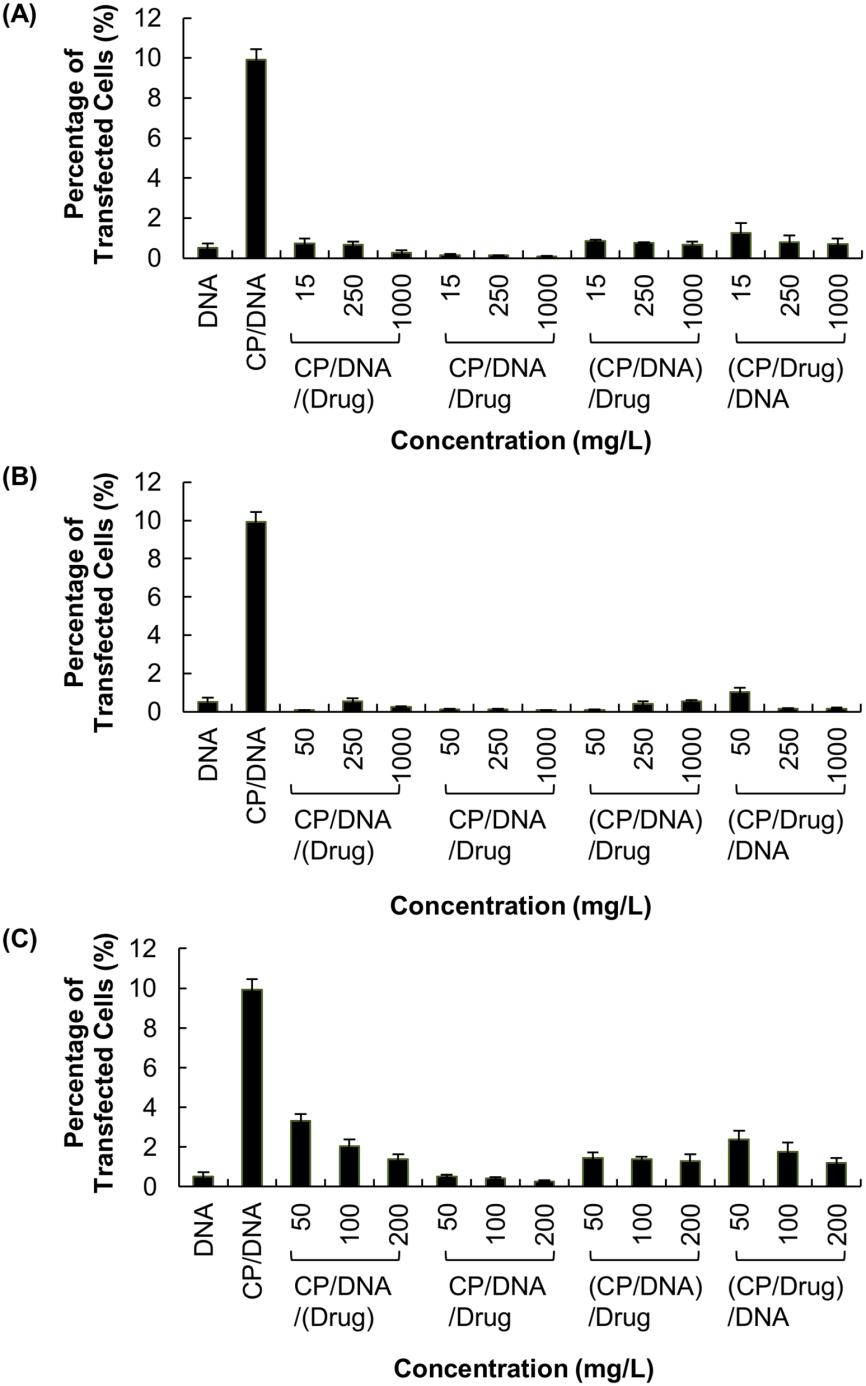
The percentage of U87 cells expressing the EGFP protein after treatment with different drug/gene co-formulations. Those co-formulations were prepared with various concentrations of (A) VM-26, (B) CDDP and (C) TMZ.

Compared to that attainable by CP/DNA polyplexes, the expression of EGFP was significantly reduced when the polyplexes were formulated with either VM-26 or CDDP (Figs 2 and 3). Contrary to VM-26 and CDDP, the interference with transfection by TMZ was relatively mild. In addition, among all gene/drug co-formulations prepared with TMZ, the formulation of “CP/DNA/Drug” led to the lowest transgene expression (Fig 3C).

### 3.2 Cytotoxicity of different gene/drug co-formulations

**Fig 4:**
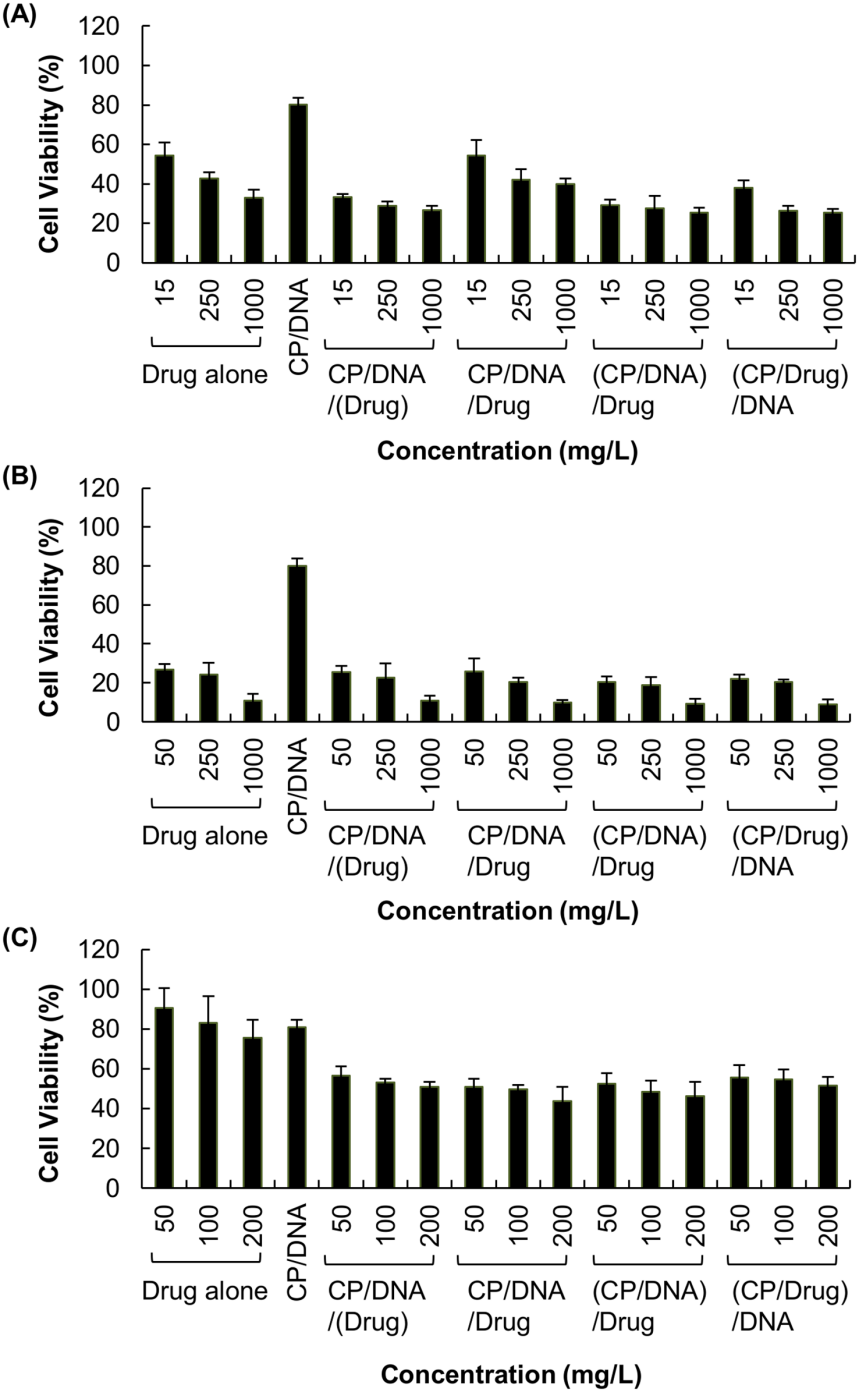
Cytotoxicity of U87 cells treated with different drug/gene co-formulations. Those co-formulations were prepared with various concentrations of (A) VM-26, (B) CDDP and (C) TMZ.

The MTT assays revealed that the cytotoxicity of polyplexes alone was negligible in U87 cells, with over 80% of cells being alive after 48 hours of post-transfection incubation (Fig 4). The cytotoxicity was substantially increased when polyplexes were formulated with chemotherapeutic drugs. As shown in Fig 4, the cell viability dropped to 30–50%, 10–30% and 40–60% after treatment with polyplexes formulated with VM-26, CDDP and TMZ, respectively.

### 3.3 Effects of drug pre-treatments

**Fig 5:**
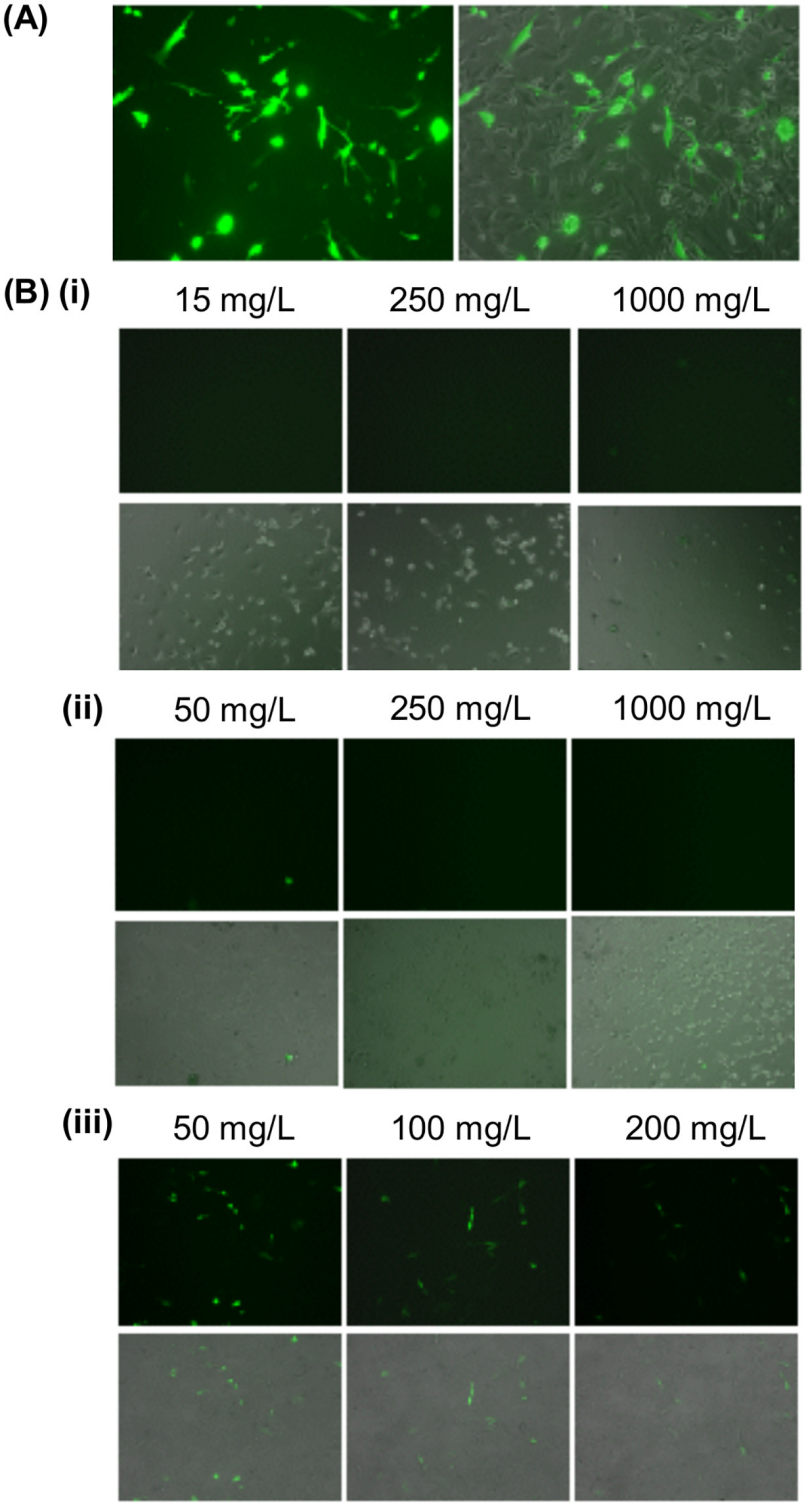
Representative EGFP fluorescence and combined phase-fluorescence micrographs of transfected U87 cells, with or without drug treatment prior to transfection. (A) Micrographs of untreated U87 cells transfected with CP/DNA polyplexes. (B) Micrographs of U87 cells pre-treated with different concentrations of (i) VM-26, (ii) CDDP and (iii) TMZ prior to transfection.

**Fig 6:**
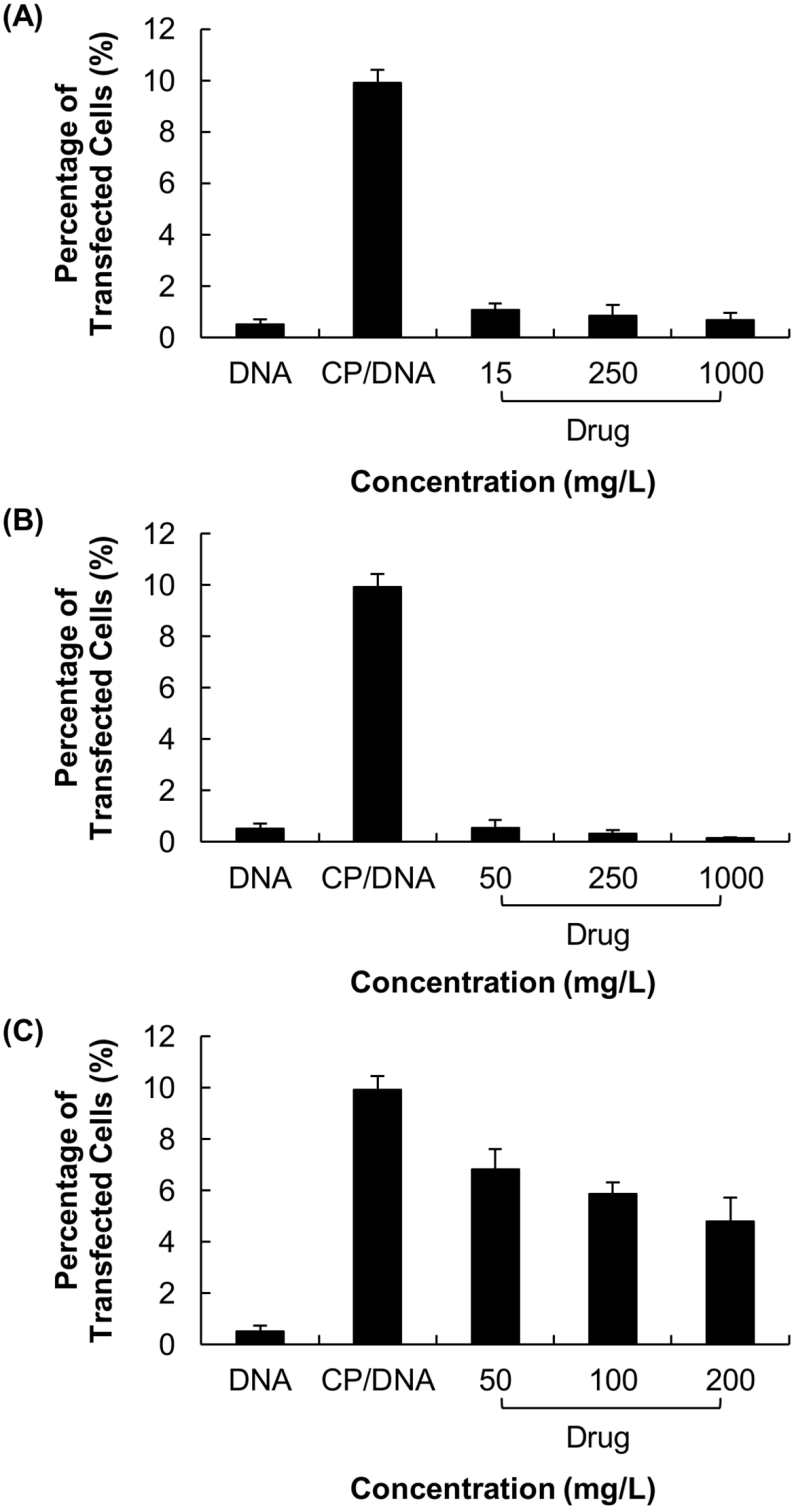
The percentage of transfected U87 cells expressing the EGFP protein. Treatments with various concentrations of (A) VM-26, (B) CDDP and (C) TMZ were applied to cells prior to transfection.

**Fig 7:**
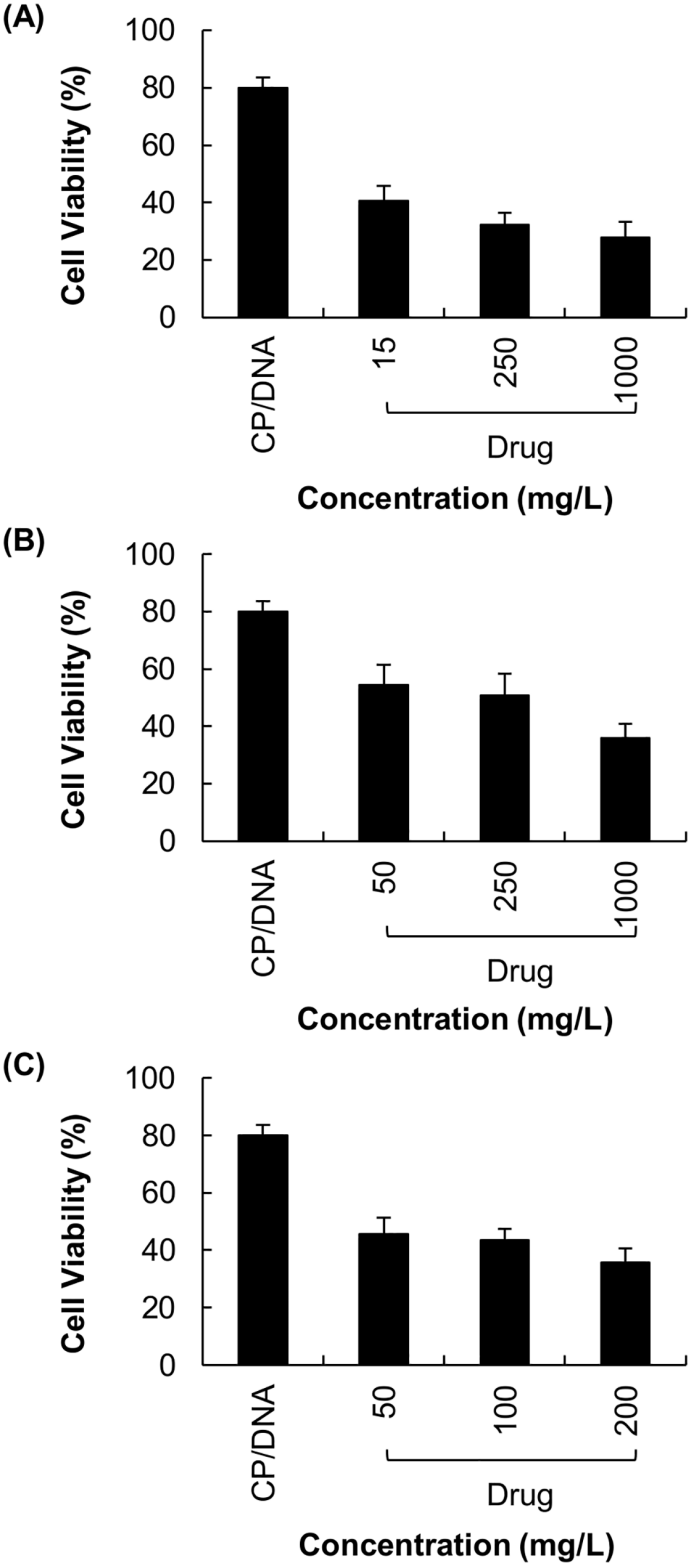
Cytotoxicity of U87 cells transfected with CP/DNA polyplexes. Treatment with various concentrations of (A) VM-26, (B) CDDP and (C) TMZ were applied to cells prior to transfection.

The effects of pre-treatment with chemotherapeutic drugs on subsequent CP-mediated transfection were presented in Figs 5–7. Compared to the control, cells pre-treated with drugs showed lower transfection efficiency (Figs 5 and 6) and higher cytotoxicity (Fig 7).

## Discussion

Combined chemo-gene therapy is one of the treatment modalities that have received considerable research attention in recent years [7,8,19]; however, the drug interference with gene delivery is ill-understood. In this study, the effects of VM-26, CDDP and TMZ on gene delivery have been studied. VM-26 is a potent drug belonging to the class of semisynthetic epipodophyllotoxin glucopyranosides (which are semisynthetic agents shown to be active against a range of leukemias and solid tumors) [20]; whereas CDDP is a common drug for treatment of malignancies including bladder, esophageal, head and neck, ovarian, and testicular cancers [21]. Finally, TMZ is a prodrug that undergoes spontaneous degradation at the physiological pH to form 5-(3-methyl-triazen-1-yl) imidazole-4-carboxamide (MITC) [22]. MITC then interferes with DNA replication by forming methyl adducts at the N3-position of adenine and the N7- and O6-positions of guanine [23]. As VM-26, CDDP and TMZ are drugs that have been extensively used in chemotherapy, they are ideal models to illustrate how gene/drug co-formulation potentially interferes with polyplex formation and transfection.

As shown in Figs 2 and 3, the expression of EGFP has been substantially reduced when the plasmid is co-delivered with VM-26 or CDDP. The reduction caused by VM-26 is thought to be due to the drug-mediated gene damage [24]. As shown by an earlier study in A549 cells [24], VM-26 introduces single-strand and double-strand DNA breaks by inhibiting the DNA ligase activity of type II topoisomerase [24]. It is expected that administration of VM-26 during CP-mediated transfection may induce breakage of the plasmid, making the transgene no longer functional for expression. Similar to VM-26, CDDP enhances the formation of DNA adducts (such as DNA monoadducts, DNA-protein cross-links, and interstrand and intrastrand DNA cross-links) [21]. Earlier experiments revealed that 1,2-intrastrand d(ApG) and d(GpG) cross-links account for 25% and 65%, respectively, of CDDP adducts formed *in vitro* [21,25,26]. By using X-ray diffraction, the two guanines in the cross-linked dinucleotide *cis*-Pt(NH_3_)_2_(d(pGpG)) were found to be destacked, with the deoxyribose sugar of the 5’-deoxyguanosine being in a C3’-endo pucker [21,27]. This suggests that CDDP can distort the DNA double helix, causing unwinding and kinking [27]. As CDDP can inhibit DNA synthesis and damage DNA by forming adducts, this explains its inhibition of transgene expression. But contrary to VM-26 and CDDP, TMZ acts mainly on DNA replication, which may not directly impact on the expression of the transgene. Therefore, the interference with transfection by TMZ is relatively mild (Figs 2 and 3). Our results show that the action mechanism of the drug is an important factor determining the degree of interference with transfection.

In addition to drug action, different methods of drug/gene co-formulation have been found to influence transfection. As shown in Fig 3C, the formulation of “CP/DNA/Drug” has led to the lowest transgene expression. It is hypothesized that due to the concomitant addition of both TMZ and DNA, the competition between TMZ and DNA for CP is the most intense. This may lead to more severe inhibition of polyplex formation. Our results suggest that the interference with transfection by drugs can be partially determined by the efficiency of polyplex formation, which varies with different methods of gene/drug co-formulation. Here it is worth noting that, due to the highly similar electrostatic nature between DNA and RNA, the electrostatic interactions of a cationic polymeric vector with DNA was principally identical to that with RNA [28]. Even though only the interference with DNA/polymer complexation has been examined in this study, the interference is expected to be applicable to RNA delivery.

In order to evaluate whether the effect of drugs on transfection is mediated solely by increasing the cell death, we have examined the viability of cells after treatment. As observed in Fig 4, the reduction in cell viability varies with different drugs. This is attributed to the variations in pharmacological profiles of different drugs. In Figs 4 and 5, the viability of cells has dropped to approximately 30–50% after treatment with various VM-26/DNA co-formulations; however, the transfection efficiency obtained has been reduced by 95%. Similar observations have also been made in CDDP/DNA co-formulations. Contrary to VM-26 and CDDP, there is no appreciable change in the cytotoxicity across all methods of TMZ/gene co-formulation adopted in this study, yet the reduction in transfection efficiency obtained varies substantially across different formulations. Such a discrepancy between cytotoxicity and transfection efficiency suggests that the drug-mediated interference with transfection is not simply due to toxicity of the drug, but the action of the drug on gene delivery also plays a significant role.

Finally, the effect of pre-treatment with chemotherapeutic drugs on the efficiency of subsequent transfection has been studied. Cells pre-treated with VM-26, CDDP and TMZ have exhibited a reduction in transfection efficiency as compared to untreated cells (Figs 5 and 6). This demonstrates that even though the drug has been removed prior to transfection, those drug molecules that have been taken up by cells still interfere with subsequent transfection. As shown in Figs 6 and 7, changes in the degree of cell viability are not correlated with the changes in the percentage of transfected cells. This implies that it is the drug interference, rather than the cytotoxicity of the drug itself, that causes a reduction in transfection efficiency.

## Conclusions

Combined chemo-gene therapy has gained increasing attention in cancer treatment since the turn of the last century; however, the interference with transfection by drugs is ill-understood. This study has employed three commonly used chemotherapeutic drugs to evaluate the effect of drug/gene co-formulation on polyplex formation and transfection. Our results evidence that the degree of drug interference varies with the mechanism of drug action as well as the method of gene/drug co-formulation. Based on the findings, though combined chemo-gene therapy has therapeutic potential, the incompatibility of chemotherapeutic drugs with gene delivery may reduce the efficiency of gene therapy in practice.
